# Available CALM (calmodulin) and autophagy: pas de deux at rest

**DOI:** 10.1080/27694127.2022.2069969

**Published:** 2022-05-01

**Authors:** Elizabeth McConnaha, Jennifer Giles, Eric Wauson, Quang-Kim Tran

**Affiliations:** Department of Physiology & Pharmacology, Des Moines University College of Osteopathic Medicine 3200 Grand Avenue, Des Moines, IA 50312, USA

**Keywords:** AMPK, autophagy, calcium, calmodulin, calmodulin antagonists, calmodulin buffering, chloroquine, lysosomal acidity

## Abstract

Macroautophagy/autophagy is a process that degrades unnecessary cellular components to maintain homeostasis. The Ca^2+^-sensing protein CALM (calmodulin) is needed for many cell functions yet is a limiting factor because of its insufficient availability for its target proteins. Although Ca^2+^ produces diverse actions in autophagy, the role of CALM in autophagy at basal conditions, when there is no external stimulation for increases in intracellular Ca^2+^, is not known. Our recent work indicates that CALM availability is critical for basal autophagy. In this punctum, we summarize our findings and discuss mechanisms whereby CALM regulates basal autophagy.

As conditions that stimulate autophagy often increase intracellular Ca^2+^ concentration ([Ca^2+^]*_i_*), the participation of CALM as the ubiquitous Ca^2+^ sensor is predicted. However, the role of CALM in basal autophagy in the absence of Ca^2+^-elevating stimuli is not known. This uncertainty is more pronounced with the fact that CALM is produced insufficiently for the numerous CALM-binding proteins (CBPs). Additionally, although some CBPs have been implicated in autophagy, changes in their direct interaction with CALM in paradigms that alter autophagy are often not shown.

In a recent work [1], we tested the role of CALM availability in basal autophagy. In competitive binding assays with CALM biosensors that binds CALM with different Ca^2+^ dependencies, chloroquine (ChQ) competes for CALM only in the presence of Ca^2+^, and thus acts as a direct antagonist of Ca^2+^-liganded CALM (Ca^2+^-CALM, Figure 1). Three other CALM antagonists, including W-7, trifluoperazine (TFP), and CGS9343b, cause accumulation of MAP1LC3/LC3 (microtubule-associated protein 1 light chain 3) and SQSTM1/p62 and inhibition of basal autophagic flux, as does ChQ. There are no additive effects between ChQ and the other CALM antagonists, suggesting that ChQ may inhibit autophagy by inhibiting CALM.

**Figure 1.**
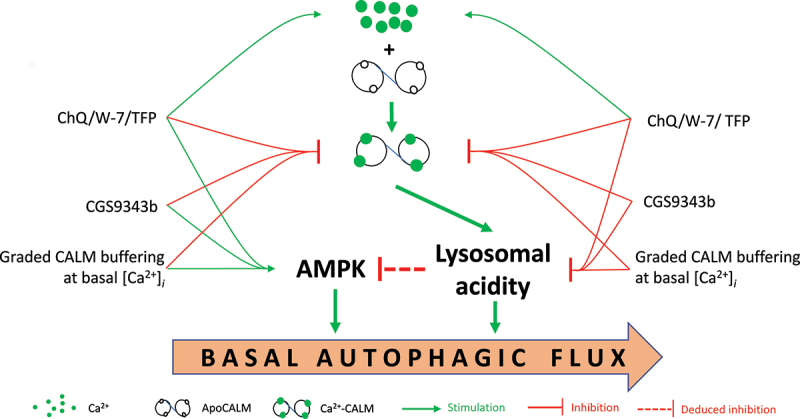
Regulation of autophagy by CALM availability. ChQ triggers increases in [Ca^2+^]*_i_* but acts as an antagonist of Ca^2+^-liganded CALM, as do CALM antagonists W-7 and TFP, whereas CGS9343b only inhibits Ca^2+^-CALM. Reduction of cytoplasmic CALM availability in basal conditions using ChQ, W-7, TFP or CGS9343b, or by buffering CALM at resting [Ca^2+^]*_i_* promotes AMPK activity but inhibits lysosomal acidification, thereby inhibiting basal autophagic flux.

How do the CALM antagonists inhibit basal autophagy? Treatment with ChQ, W-7, TFP or CGS9343b does not affect the activity of MTOR (mechanistic target of rapamycin kinase) but activates AMP-activated protein kinase (AMPK), whose activation would be predicted to promote autophagy. However, all four reagents significantly increase lysosomal pH, consistent with their effects to inhibit basal autophagic flux (Figure 1). As formation of Ca^2+^-CALM complexes that activate CBPs depends on both [Ca^2+^] and available CALM, the antagonists might inhibit autophagy by affecting either factor. ChQ, W-7 and TFP mobilize Ca^2+^ from both intra- and extracellular sources, whereas CGS9343b has no effect, which is further confirmed by the absence of Ca^2+^ extrusion upon extracellular Ca^2+^ removal following prolonged CGS9343b treatment. Interestingly, chelating basal intracellular Ca^2+^ strongly prevents the effects of the CALM antagonists to cause accumulation of MAP1LC3/LC3, including that of CGS9343b. These disparate effects suggest that these reagents inhibit autophagy by inhibiting CALM, and that basal [Ca^2+^]*_i_* is sufficient to support CALM-dependent regulation of autophagy.

Does CALM regulate autophagy by acting in the cytosol or in an organelle(s)? And how can the possibility be ruled out that the CALM antagonists inhibit autophagy by entering lysosomes and chemically disturb lumenal acidity independently of CALM antagonism? These related questions cannot be answered by silencing or overexpressing CALM, given that basal organellar Ca^2+^ levels are high, and that CALM can translocate between the cytosol and organelles. We thus tested the effects of “graded” cytoplasmic CALM buffering on MAP1LC3/LC3 level, activities of MTOR and AMPK, and lysosomal pH at basal condition and following amino acid removal. Two high-affinity CBPs were overexpressed that have different cytoplasmic localizations and numbers of CALM-binding domains (CBD) per monomer. The first is a plasma membrane-bound NOS3/eNOS mutant (NOS3^S1179D^, 1 CBD/monomer) that binds CALM with sub-nanomolar K*_d_* at resting [Ca^2+^] and is tagged C-terminally to DsRed2, whose fluorescence enables recognition of transfected and non-transfected cells in the same microscopy fields for reliable comparison. CALM buffering was verified by simultaneously measuring free [Ca^2+^] and [Ca^2+^-CALM], and nitric oxide production was prevented pharmacologically. The second is a cytoplasmic fusion between a CALM biosensor (BSCALM_2,_ 1.4-nM K*_CALM_*) tagged N-terminally to NOS3 (0.2-nM K*_CALM_*). BSCALM_2_-eNOS has 2 CBDs/monomer and combined Ca^2+^ sensitivities for CALM binding from resting [Ca^2+^]. Buffering sub-plasmalemmal cytoplasmic CALM with NOS3^S1179D^-DsRed2 significantly increases the LysoSensor ratio by ~20%, whereas a non-binding mutant (NOS3^T497D^-DsRed2) does not. Enhanced cytoplasmic CALM buffering at rest with BSCALM_2_-eNOS increases the LysoSensor ratio by ~224% and significantly increases AMPK activity but does not affect MTOR activity. Amino acid removal predictably reduces lysosomal pH, reduces MTOR activity and increases AMPK activity; however, the differences caused by CALM buffering remain.

Several points are noteworthy from these studies (Figure 1). First, the effects of ChQ as a CALM antagonist that triggers Ca^2+^ signals are worth considering in autophagy studies. Second, lysosomal acidity is strongly regulated by cytoplasmic CALM availability. Although the V-ATPase binds CALM, a CALM binding-deficient V-ATPase mutant does not affect lysosomal acidity. The CBP(s) involved in this process thus remain(s) unclear. Third, reduced CALM availability invariably couples with enhanced AMPK activity but unchanged MTOR activity. This obviously cannot be explained via known CALM-dependent processes upstream of AMPK. More likely, a link between lysosomal acidification and AMPK activity is deduced (Figure 1), which is supported by reports that V-ATPase inhibition promotes AMPK activity. Although many questions remain, our work implicates available CALM in an intricate pas de deux with basal, and perhaps starvation-induced, autophagy, and suggests that conditions that alter CALM availability will significantly affect this process.
